# Gluten-Free Diet and Its ‘Cousins’ in Irritable Bowel Syndrome

**DOI:** 10.3390/nu10111727

**Published:** 2018-11-11

**Authors:** Anupam Rej, David Surendran Sanders

**Affiliations:** 1 Academic Unit of Gastroenterology, Royal Hallamshire Hospital, Sheffield Teaching Hospital NHS Foundation Trust, Sheffield S10 2JF, UK; anupam.rej@sth.nhs.uk; 2 Academic Unit of Gastroenterology, Department of Infection, Immunity and Cardiovascular Disease, University of Sheffield, Sheffield, S10 2RX, UK

**Keywords:** non-coeliac gluten sensitivity, gluten, wheat, low FODMAP diet, irritable bowel syndrome

## Abstract

Functional disorders are common, with irritable bowel syndrome (IBS) being the commonest and most extensively evaluated functional bowel disorder. It is therefore paramount that effective therapies are available to treat this common condition. Diet appears to play a pivotal role in symptom generation in IBS, with a recent interest in the role of dietary therapies in IBS. Over the last decade, there has been a substantial increase in awareness of the gluten-free diet (GFD), with a recent focus of the role of a GFD in IBS. There appears to be emerging evidence for the use of a GFD in IBS, with studies demonstrating the induction of symptoms following gluten in patients with IBS. However, there are questions with regards to which components of wheat lead to symptom generation, as well as the effect of a GFD on nutritional status, gut microbiota and long-term outcomes. Further studies are required, although the design of dietary studies remain challenging. The implementation of a GFD should be performed by a dietitian with a specialist interest in IBS, which could be achieved via the delivery of group sessions.

## 1. Introduction

Functional disorders are common, with the Rome IV guidelines classifying these disorders into oesophageal, gastroduodenal, bowel, centrally mediated, anorectal, gallbladder, and sphincter of Oddi disorders [1]. The commonest and most extensively evaluated functional bowel disorder is irritable bowel syndrome (IBS), with a reported global pooled prevalence of 11 percent [2]. IBS can be classified into diarrhoea-predominant (IBS-D), constipation-predominant (IBS-C), mixed (IBS-M) and unclassified (IBS-U) [1]. The pathophysiology of IBS is not fully understood, but several pathophysiological mechanisms have been proposed, including visceral hypersensitivity, inflammation, increased intestinal mucosal permeability, and genetic and psychological factors [3,4]. The impact of IBS can lead to a significant impact on sufferers, with a reduced quality of life, increased time off work and greater utilisation of healthcare [5]. It is also associated with several other conditions such as depression, fibromyalgia, chronic fatigue disorder, and temporomandibular joint disorder [6]. As a result of this, it is paramount that there are effective treatments for this common condition.

Despite many individuals using medication for the treatment of functional disorders, up to two-thirds of individuals with functional disorders also use diet or dietary supplements as a therapy [7]. A large proportion of individuals with IBS note that they have dietary triggers, with up to two-thirds of individuals noting the induction of symptoms after ingestion of food [8,9,10]. As a result of this, there has been great interest in the role of dietary therapies in IBS, with a focus recently in a low fermentable oligo-, di-, and mono-saccharides and polyols (FODMAPs) diet, wheat free diet (WFD) and gluten-free diet (GFD).

There has been an increase in the awareness of the GFD over the last decade [11], with the gluten-free food industry continuing to rise, with more than $15 billion dollars spent in the USA in 2016 [12]. Whilst GFDs are known as the mainstay of treatment for people with an established diagnosis of coeliac disease [13], we will explore the emerging evidence for this dietary therapy in individuals with IBS.

## 2. Wheat Free Diet

Gluten is found in the endosperm of grass-related grains, including wheat, barley, and rye. In view of this, it is important to explore the role of a WFD in IBS as many individuals consuming a GFD maybe avoiding wheat.

Wheat avoidance is common, with a cross-sectional population survey in Australia reporting wheat avoidance in approximately 10% of individuals [14]. A significant proportion of individuals with IBS who reduce wheat consumption may have a wheat sensitivity. A large study [15], in which 920 patients with IBS who had a self-reduced wheat consumption, underwent an elimination diet for 4 weeks, subsequently followed by a double-blind placebo-controlled (DBPC) challenge. Out of these patients, 276 patients (30%) were suffering from a wheat sensitivity, as they were identified as being asymptomatic on an elimination diet, followed by symptoms during the DBPC challenge.

The same group evaluated the same cohort of patients in a prospective study [16], with a median follow up of 99 months in 200 patients. A total of 148 patients (74%) were still following a wheat free diet at follow up, and 127 patients (64%) were on a strict gluten-free diet. A total of 22 of these patients were randomised to the DBPC wheat re-challenge. A total of 20 of the 22 patients (91%) still reacted to wheat. This suggests that even in the long term, a subgroup of patients with IBS is likely to have a persistent sensitivity to wheat.

Confocal endomicroscopy has shown immediate and dramatic mucosal responses to wheat as an antigen [17]. Another study with 80 participants who reported symptoms related to wheat, demonstrated systemic immune activation and compromised intestinal epithelial barrier integrity in these individuals [18]. This may provide a morphological basis for wheat causing symptoms in individuals with IBS. There is currently little data known on the long-term risks of a wheat free diet in patients with IBS, with studies required to assess this.

## 3. Gluten-Free Diet

Many individuals note symptoms following the ingestion of gluten. This has led to the term of non-coeliac gluten sensitivity (NCGS), being described as early as the 1980s [19]. The mechanism of induction of symptoms in individuals is unclear, but it has been suggested that gluten proteins may be insufficiently degraded by gut proteases, which could lead to an innate immune response [20]. However, further research is required to elucidate the mechanism.

Gluten has been demonstrated to generate symptoms in individuals with IBS [21]. A DBPC trial in 34 patients with a diagnosis of IBS who had improved on a GFD were given either gluten or placebo in the form of two bread slices plus one muffin per day, with a GFD for up to 6-weeks. The study demonstrated worsening symptoms on the visual analogue scale (VAS) for overall symptoms (*p* = 0.047), pain (*p* = 0.016), bloating (*p* = 0.016), stool consistency (*p* = 0.024), and tiredness (*p* = 0.001) with gluten. The same group later performed a study demonstrating no effects of gluten in patients with NCGS and IBS [22], in contrast to their previous study. This double-blind crossover trial in 37 subjects with NCGS and IBS involved placing participants on a 2-week diet of reduced FODMAPs, followed by the random allocation to high-gluten (16 g gluten/day), low-gluten (2 g gluten/day and 14 g whey protein/day), or control (16 g whey protein/day) diets for 1 week, followed by a washout period of at least 2 weeks. The authors concluded that there were no dose-dependent effects of gluten on patients placed on a low FODMAP diet. However, the findings of this study may be explained by the study design and participants. As participants knew they were going to receive either high-gluten, low-gluten, or control, there may have been an anticipatory nocebo response which may have accounted for these findings. Secondly, participants at baseline had a high VAS, which may not be truly representative of this group.

There have also been other double-blind placebo-controlled trials evaluating the effect of gluten on IBS. A study in 148 patients with IBS involved commencing individuals on a GFD, followed by packages containing powdered gluten or gluten-free powder [23]. Symptomatic improvement was different in the gluten-containing group in comparison to the placebo group, with symptoms being controlled in 25.7% in the gluten-containing group, compared to 83.8% in the placebo group. Another DBPC trial in India [24] showed similar findings. A total of 60 patients with IBS completed this study, in which participants underwent a GFD for 4 weeks, followed by a re-challenge of placebo (gluten-free breads) or gluten (whole cereal breads). The study demonstrated that participants in the gluten intervention group scored higher in terms of abdominal pain, bloating, and tiredness (*p* < 0.05).

Randomised controlled trials (RCT) have demonstrated the benefit of a GFD. An RCT [25] in 45 patients with IBS-D, in which participants were allocated to either a 4 week trial of a GFD or gluten-containing diet, demonstrated an increase in bowel movements per day in those on a gluten-containing diet (*p* = 0.04). Interestingly, individuals taking a gluten-containing diet were noted to be associated with a higher small bowel permeability, with small bowel permeability greater in HLA DQ2/8 positive than negative patients (*p* = 0.018). A GFD was noted to have a greater effect of bowel movements per day in HLA DQ2/8 positive than negative patients (*p* = 0.019). It is, therefore, possible that gluten affects the bowel barrier function on IBS, with HLA DQ2/8 being a susceptible factor. A prospective study [26] in 41 patients also demonstrated a clinical response (reduction in IBS-Symptom Severity Score from 286 to 131, *p* < 0.001) in individuals with IBS-D placed on a 6 week GFD. Interestingly, from this study, 21 out of the 29 individuals (72%) in the study planned to continue this diet in the long term, with individuals noted to still be on the diet at a mean of 18 months.

The majority of patients taking a GFD in IBS appear to be adherent to the diet. A study [27] in 35 patients with IBS noted that in those individuals who responded to a GFD, 64% (7 out of 11 patients) were still adherent. This data are similar to patients with coeliac disease, where full adherence has been reported at 65 percent and partial adherence at 31 percent [28]. This may suggest the ease of implementation of a GFD in IBS. Additionally, the GFD is an exclusion diet, which is defined as the exclusion of one or two foods from the diet, in comparison to an elimination diet, which involves the removal of a selection of foods [29]. This may help with ease of implementation of the GFD in comparison to elimination diets, such as the low FODMAP diet. However, there have been no direct comparisons in the literature between the low FODMAP diet and GFD with regards to ease of implementation.

Individuals who maintain a GFD have the option of purchasing specialised gluten-free products. For individuals with coeliac disease consuming a GFD, it has been demonstrated that the majority of individuals purchase gluten-free products [30]. A study in the UK demonstrated that gluten-free foods were 4 times more expensive than non-gluten-free alternatives (*p* < 0.0001), with regular and quality supermarkets stocking a median of 22 items, in comparison to nil in budget supermarkets [31]. This may suggest that cost may limit the purchase of specialised gluten-products, especially for individuals from lower socioeconomic classes. However, it is important to note that alternatively there are naturally occurring foods free of gluten, which do not require the purchase of specialist products to maintain a GFD. Therefore, it is uncertain whether a GFD is more expensive to implement than a standard diet, and there is no literature to date on this in patients with IBS.

Recently a combination of the low FODMAP diet and GFD (LF-GFD) demonstrated benefit in patients with coeliac disease and co-existing functional symptoms [32]. This randomised double-blind study recruited patients with coeliac disease on a GFD for at least one year, with a negative plasma tissue transglutaminase (TTG) value with IBS symptoms fulfilling the Rome III criteria. A total of 50 participants were randomly allocated to an LF-GFD or GFD only. Participants received a structured dietary plan from a nutritionist for a 21-day period. A significant reduction in the VAS for abdominal pain was noted in the LF-GFD group versus the regular GFD group (*p* < 0.01). The general well-being increased in both groups, although a higher improvement was noted in the LF-GFD combination group (*p* = 0.03) [32]. Further studies are required to assess whether this could be an effective therapy for individuals with coeliac disease and IBS, as well as long-term studies being required.

## 4. Unanswered Questions

Despite the growing evidence for a GFD, a number of questions remain. Little is known about the potential effect of nutritional deficiencies in patients with IBS undergoing a GFD, with data being extrapolated from individuals on a GFD as a result of coeliac disease. A prospective validated 5-day food diary [33], in which 139 patients with coeliac disease were invited to fill, demonstrated similar intake of nutrients and energy to comparator populations, but a higher proportion of carbohydrate intake was obtained from non-milk extrinsic sugars and intakes of non-starch polysaccharides were low. Another study in 47 individuals with coeliac disease on a GFD, estimated three-day food records. Lower than recommended intakes of fibre and calcium in men and women was noted [34]. A GFD has been demonstrated to be poor in alimentary fibre, as well as micronutrient deficiencies being noted [35]. Whilst macronutrient and micronutrient deficiencies have been demonstrated on a GFD, these may not necessarily be a result of the GFD itself. It may be that the changes seen are reflective of overall community dietary habits and pre-existing individual eating habits, rather than the GFD alone [36,37]. For example, a cross-sectional population-based study in the UK [38] demonstrated that over 95% of men and women were not adherent to fibre recommendations. A significant reduction in energy intake has also been demonstrated in individuals following traditional dietary advice [39]. Additionally, it is unknown whether individuals with IBS will have the same macro- and micro-nutrient deficiencies as individuals on a GFD for coeliac disease, and this may differ on the level of gluten restriction implemented by individuals.

As can be seen, macro- and micronutrient deficiencies can occur with any diet, and this highlights the importance of dietetic involvement in the implementation of dietary therapies and is supported by other reviews [40,41]. It has been shown that whilst the vast majority of physicians gave patients advice about the improvement of diet or dietary habit for IBS [42], only a minority would recommend referral to a specialist dietitian [43]. The GFD for IBS should be implemented by a dietitian with a specialist interest in IBS, with physicians referring to dietitians for assessment. This is on the basis that the evidence base for dietary therapies for IBS has been derived from dietitian-led studies rather than physician-led dietary advice [41]. Whilst a GFD diet may be beneficial for some individuals with IBS, it is important that the most appropriate dietary therapy is identified by a dietitian through a detailed history involving the patient. This is important as there is evidence also for the use of a low FODMAP diet and WFD [44], with the NICE [45] and BDA [46] guidelines being recommended as first-line dietary therapies for IBS currently. The implementation of dietary therapies can also lead to the development of obsessive behaviours and orthorexia nervosa [47], highlighting the need for dietetic input to prevent this. Whilst a dietitian-led approach is advised, this is likely to lead to a strain on existing resources though, with the need for novel methods to increase efficiency if able. In IBS patients undergoing a low FODMAP diet (*n* = 364), dietitian-led group education has been demonstrated to be clinically effective [48], and this method could potentially be a cost-effective way to implement a GFD to patients with IBS. It is important to note, however, that there is little data evaluating group therapies versus one-to-one for a GFD in IBS to date.

Studies have explored the effect of the gut microbiota in individuals on a GFD in healthy individuals, as well as in individuals with coeliac disease [49,50,51,52]. A study in ten healthy individuals [52] using faecal samples assessed the effect of a GFD over 4 weeks on gut composition and microbiota. Bacterial populations regarded to be beneficial for health such as Bifidobacterium proportions were shown to decrease after the consumption of a GFD, raising potential concerns of a GFD. Additionally, Faecalibacterium prausnitzii proportions were shown to decrease after the consumption of a GFD in this study, with these bacteria known to be an important butyrate-producer in the colon, with butyrate being known as a key modulator of colonic health [53]. It has also been suggested that taxon-specific shifts as a result of the GFD may explain the benefits of a GFD seen in patients with IBS. A study [49] in 21 healthy individuals consuming a GFD over 4 weeks demonstrated stable inter-individual variation in the gut microbiota, with a shift of taxon-specific differences, most marked with Veillonellaceae. Veillonellaceae is considered to be a pro-inflammatory family of bacteria, and a decrease in its abundance on the GFD could be one of the mediators of the benefit observed in patients with IBS on a GFD. The GFD appears to affect species particularly involved in starch and carbohydrate metabolisms [49]. It is unclear whether this would occur in a population of IBS patients and, therefore, studies are required to assess this. Assessment of the effect of a GFD on the gut microbiota in both the short and long term is required in patients with IBS. Currently, the role of the GFD on the gut microbiota is unclear. The changes seen could be due to a GFD, or they could be due to other dietary alterations made whilst on a GFD. In the studies mentioned above [49,52], faecal flora was analysed. The faecal flora is highly organised and spatially organised [54], leading to an uneven distribution in stool samples [55], which suggests that faecal samples are unlikely to be truly representative of the gut microbiota. Sample sizes used in the studies have been small and individuals tend to be unique in terms of their gut microbiota [55]. It is also important to note that these studies focussed on the gut bacteriome, rather than the virome and mycobiome and, therefore, may not be truly representative of the entire microbiota. Further studies are required to explore the effect of the GFD on the microbiome, with the assessment of both short-term and long-term changes.

A recent systematic review and meta-analysis assessing the efficacy of a GFD in IBS [40] concluded that there was insufficient evidence to recommend a GFD to reduce IBS symptoms. Participants in the two studies [21,23] included in the review were intolerant of gluten in addition to IBS, which may suggest that these studies were not fully representative of the entire IBS population. As only two RCTs were deemed suitable for review, this led to a small sample size (*n* = 111), giving insufficient evidence for the reviewers to recommend this diet. However, as seen in Table 1, there have been a number of studies in addition to these assessing the role of the GFD in IBS [21,23,24,25,26,27,56,57,58]. It would be difficult to include all these studies in a meta-analysis as it would be difficult to combine the data from all these studies due to the heterogeneity of these studies. Studies assessing the GFD in IBS have used different methods of delivering a GFD, such as using feeding studies in some trials and dietary advice in others. In feeding studies, different doses of gluten have also been used. Different primary outcomes have been assessed, different population groups have been enrolled, as well as different study durations. As can be seen in Table 1, the studies have been performed in a wide variety of geographic locations which may lead to divergent results as different geographic locations may employ the GFD differently, which also may potentially have an impact on their resident gut microbiota [55]. The design of these studies is important as this may result in different outcomes. For example, the studies by Biesiekierski and colleagues [21,22], assessing the role of a GFD in IBS, led to different outcomes as mentioned earlier, which could be attributed to study design.

Studies have focussed on patient symptoms to determine response using validated questionnaires such as the IBS-SSS [59], rather than objective biomarkers. Currently, the evidence does not suggest one biomarker in IBS, but rather a panel of biomarkers [60]. Currently, even using a panel of biomarkers has a poor sensitivity and specificity and, therefore, has limited the use of biomarkers to assess response to dietary therapies in IBS [60,61]. Therefore, further research is required in this area before objective biomarkers can be used to assess response to therapies in IBS. It appears from Table 1 that there are several individual trials demonstrating the benefit of a GFD in IBS, demonstrating the growing evidence base for its use in IBS.

There have been a relatively smaller number of patients recruited to studies assessing the GFD in IBS. This is likely to be an issue for dietary studies in general, with a lack of pharmaceutical support for dietary therapy trials in comparison to pharmaceutical trials, as well as IBS not being a priority area for research [40]. Significant challenges remain, with a lack of guidelines for dietary trials, unlike drug trials which are closely regulated [62]. Issues also remain with regards to blinding, for example, as the GFD is well known to the general public, with up to 5 percent of individuals taking a GFD on their own volition [63,64]. In addition, other challenges remain in designing dietary trials, including difficulties in manipulating the diet and the adherence and modification of dietary habits. It is also difficult to practically implement the findings from dietary trials to the real world [65].

It is unclear which component of wheat leads to symptom generation. Several components have been suggested as the causal agent, including gluten, alpha-amylase trypsin inhibitors (ATIs), wheat germ agglutinins (WGAs), and fructans, which are part of the low FODMAP diet [66]. Studies have been performed to try to elucidate the pathophysiological mechanisms of these components in symptom generation. Gluten has been demonstrated to alter bowel barrier functions in patients with IBS. The expression of tight junction proteins (ZO-1, occludin, and claudin-1) have been demonstrated to be significantly lower in the colonic mucosa of individuals on a gluten-containing diet, especially in individuals who are HLA DQ2/ 8 positive [25]. Tight junction proteins, claudin-2, 8 and 15, as well as myosin light chain kinase (MLCK)-myosin II regulatory light chain (MLC) pathway have been demonstrated to be important in intestinal physiology and barrier function, regulating paracellular permeability. A study [67] evaluating biopsies from 27 patients with IBS-D demonstrated alterations in MLC phosphorylation and claudin-15 and claudin-2 expression with gluten with intestinal permeability changes. This also could potentially explain permeability responses to gluten challenge in patients with IBS [67]. ATIs have been demonstrated to be strong induces of the innate immune responses, in vitro and in vivo, via the activation of the toll-like receptor 4, with the release of pro-inflammatory cytokines leading to intestinal inflammation [68]. WGA is the best-studied cereal grain lectin. When delivered in vitro, WGAs have been demonstrated to stimulate monocytes and macrophages, which have the ability to initiate and maintain inflammatory responses [69]. WGA has been demonstrated to affect enterocyte permeability in vitro. However, it is important to note that human data demonstrating WGA on inflammatory markers are lacking [69]. FODMAPs are short-chain carbohydrates which are rapidly fermentable and poorly absorbed, increasing the small bowel water content, passing unaltered into the colon, where they are rapidly fermented, generating gas and distention [70]. Similar physiological responses to FODMAPs has been demonstrated in both healthy individuals and patients with IBS, indicating that colonic hypersensitivity to distention in patients with IBS is likely to be the pathophysiological mechanism [71]. FODMAPs are considered to be beneficial to epithelial cell integrity and health [72].

A recent study has suggested that fructans rather than gluten are responsible for symptoms seen in patients with IBS. The study [73] in 59 individuals who had already self-instituted a GFD involved a double-blind crossover challenge, in which individuals were randomly assigned to diets containing fructans, placebo, and gluten for 7 days, followed by a minimum 7 day washout period. The overall gastrointestinal symptom rating scale (GSRS) score for participants consuming fructans was significantly higher than those consuming gluten (*p* = 0.049).

**Figure 1:**
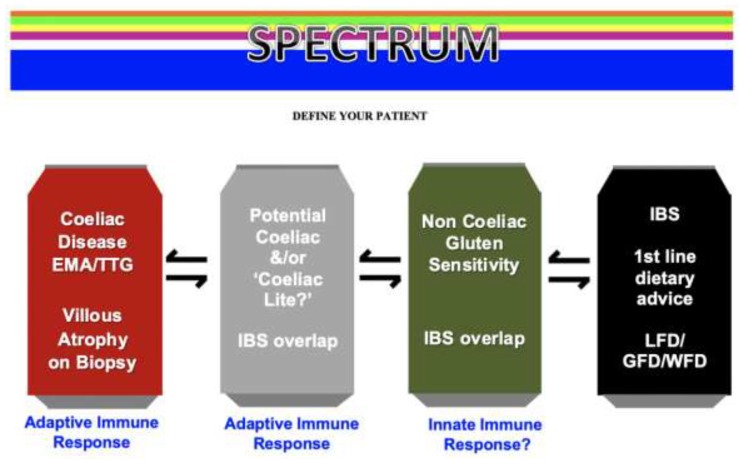
The spectrum of Gluten Related Disorders. EMA: endomysial antibodies; TTG: tissue transglutaminase; IBS: irritable bowel syndrome; LFD: low fermentable oligo-, di-, and mono- saccharides, and polyols diet; GFD: gluten-free diet; WFD; wheat-free diet.

It is likely that there is a significant overlap between dietary therapies used in IBS including the GFD, WFD, and low FODMAP diet. They are likely to be ‘dietary cousins’, with each diet being needed to be tailored to the individual patient after a detailed assessment by a dietitian. There appears to be a spectrum of gluten-related disorders, as seen in Figure 1.

It is important to note that there are several other dietary therapies which are being explored in patients with IBS. As the evidence is growing for gluten in generating symptoms in IBS, it is possible to hypothesise that other foods may also contain harmful molecules for patients with IBS. Some dietary therapies have focussed on diets with a primary focus on bioactive food molecules, such as the low capsaicin diet, low amine/histamine diet, and low food chemical diet [47]. Additionally, there are diets in addition to the low FODMAP diet focussing on the carbohydrates like the specific carbohydrate diet and paleo diet. Likewise, there are diets in addition to the GFD focussing on proteins, such as reduced resistant protein diet. However, to date, there is little evidence for the use of these diets in patients with IBS [47].

## 5. Conclusions

There appears to be emerging evidence for the use of a GFD in IBS. A number of unanswered questions remain, including the effect on the gut microbiota in both the short and long term, as well as the effect on short- and long-term nutritional adequacy. This dietary therapy should be implemented by a dietitian with a specialist interest in IBS, which could be done through group clinics, although research is required to validate this. There is likely to be an overlap with other dietary therapies such as the low FODMAP diet, with which component of wheat leading to the induction of symptoms still being unclear. Further research is required on the use of a GFD in IBS, but dietary studies are likely to be challenging, with blinding and funding being some of the issues.

## Figures and Tables

**Table 1:** The studies evaluating gluten and wheat in Irritable Bowel Syndrome.

Lead Author for Study	Year	Location		Study Design	Study Duration	Total Number of Participants in Study	Intervention	Outcome
Wahnschaffe [56]	2001	Germany		Prospective study	6 months	102 patients with IBS-D	Gluten-free diet	Improvement in stool frequency in HLA DQ2/DQ8 positive subjects
Biesiekierski [21]	2011	Australia		DBPC trial	6 weeks	34 patients with IBS symptomatically controlled on gluten-free diet	Placebo *n* = 15 Gluten *n* = 19	Worsening of overall symptoms on VAS (*p* = 0.047), as well as pain (*p* = 0.016), bloating (*p* = 0.016), stool consistency (*p* = 0.024) and tiredness (*p* = 0.001) following gluten introduction
Carroccio [15]	2012	Italy		Crossover DBPC trial	5 weeks	276 patients with IBS identified as having wheat sensitivity	All participants received wheat or xylose (placebo) capsules	Increase in overall symptoms following the introduction of wheat (*p* < 0.0001)
Vazquez-Roque [25]	2013	USA		Randomised controlled trial	4 weeks	45 patients with IBS-D	Gluten-containing diet *n* = 22 Gluten-free diet *n* = 23	More bowel movements per day on a gluten-containing diet (*p* = 0.04)
Biesiekierski [22]	2013	Australia		Crossover DBPC trial	2-week run in of low FODMAPs then 1 week of high-gluten, low gluten, or placebo for 1 week followed by 2-week washout period	37 patients with IBS and NCGS	All participants received high gluten, low gluten or placebo	No effect of gluten on GI symptoms
Di Sabatino [57]	2015	Italy		Crossover DBPC trial	5 weeks	59 patients with self-reported NCGS	Gluten *n* = 30 Placebo *n* = 29	Intake of gluten significantly increased overall symptoms compared to placebo (*p* = 0.034)
Shahbazkhani [23]	2015	Iran		DBPC trial	6 weeks	72 patients with IBS on GFD	Placebo *n* = 37 Gluten *n* = 35	Statistically significant worsening of symptoms in the gluten-containing group versus placebo (*p* < 0.001)
Aziz [26]	2016	UK		Prospective study	6 weeks	41 patients with IBS-D	All participants received GFD	Reduction in mean IBS Symptom Severity Score from 286 to 131 (*p* < 0.001)
Zanwar [24]	2016	India		DBPC trial	4 weeks	60 patients with IBS who responded to GFD	Placebo *n* = 30 Gluten *n* = 30	Worsening of symptoms following intake of gluten (*p* < 0.05)
Elli [58]	2016	Italy		Crossover DBPC trial	3-week run in of GFD, followed by randomisation to gluten or placebo for 1 week followed by 1 week washout period	140 patients with functional symptoms (77 patients with IBS)	Placebo *n* = 48 Gluten *n* = 50	14% of patients who responded to gluten withdrawal noted to have symptomatic relapse during gluten challenge
Barmeyer [27]	2017	Germany		Prospective study	12 months	35 patients with IBS-D/M	Gluten-free diet	34% of participants (*n* = 12) responded to GFD

DBPC: double-blind placebo-controlled; IBS: irritable bowel syndrome; VAS: visual analogue scale; IBS-D, diarrhoea-predominant IBS; NCGS: non-coeliac gluten sensitivity; GFD: gluten-free diet; FODMAPs: fermentable oligo-, di-, and mono-saccharides, and polyols.
